# Is there an association between PEPFAR funding and improvement in national health indicators in Africa? A retrospective study

**DOI:** 10.1186/1758-2652-13-21

**Published:** 2010-06-12

**Authors:** Herbert C Duber, Thomas J Coates, Greg Szekeras, Amy H Kaji, Roger J Lewis

**Affiliations:** 1 Department of Emergency Medicine, Harbor-UCLA Medical Center, Torrance, California, USA; 2 Los Angeles Biomedical Research Institute, Torrance, California, USA; 3 Department of Medicine, David Geffen School of Medicine at UCLA, Los Angeles, California, USA

## Abstract

**Background:**

The US President's Emergency Plan for AIDS Relief (PEPFAR) was reauthorized in June 2008 with a three-fold increase in funds, and a broader, more explicit mandate to improve health in the low- and middle-income countries that it funded. However, the ability of a disease-specific, or vertical, programme to have a spill-over effect and improve health outcomes has been questioned. In this study, we sought to examine associations between being designated as a PEPFAR focus country (and receiving increased PEPFAR funding) and non-HIV-specific health outcomes in the World Health Organization (WHO) Africa Region, the area most affected by the HIV/AIDS epidemic.

**Methods:**

A retrospective analysis of publicly available health outcomes data published by the World Health Organization was performed for all countries in the WHO Africa Region. Fractional changes in health indicators between 2000 and 2006 were calculated, and PEPFAR focus and non-focus countries were then compared.

**Results:**

Overall, countries in the WHO Africa Region showed a small worsening in health outcomes status when all indicators were analyzed together and weighted equally. However, more health indicators improved than worsened over this six-year period. A comparison of PEPFAR focus and non-focus countries found no significant difference in the fractional change among 13 of 14 health indicators during the study period.

**Conclusions:**

This study suggests that vertical programmes, even one that is the scale of PEPFAR, may have little or no impact on health outcomes not explicitly targeted.

## Background

The HIV/AIDS epidemic has taken a substantial toll worldwide. Yet nowhere is the effect of this disease felt more deeply than in sub-Saharan Africa, where nearly two-thirds of the estimated 33 million people worldwide infected with HIV live [1]. As part of the global response to HIV, there has been a significant increase in funding to low- and middle-income countries to strengthen treatment, prevention and research programmes [2,3]. Nearly US$10 billion in funding was earmarked in 2008 for HIV/AIDS in low- and middle-income countries, representing an approximate 20-fold increase from a decade ago [2].

The largest effort by a single government to combat HIV/AIDS, the President's Emergency Plan for AIDS Relief (PEPFAR) was first authorized by the United States Congress and signed into law in 2003 with a budget of US$15 billion over five years. Fifteen focus countries, 12 of them in sub-Saharan Africa, were chosen as beneficiaries of two-thirds of the PEPFAR funds [4]. PEPFAR's five-year performance targets for the focus countries were to support prevention of seven million HIV infections, treat two million people with HIV/AIDS with antiretroviral therapy, and care for 10 million people infected with and affected by HIV/AIDS, including orphans and other vulnerable children [5].

In its 2008 report to Congress, the Office of the United States Global AIDS Coordinator (OGAC) reported that many of these goals were close to being met [6]. On 30 June 2008, the President of the United States, with the consent of Congress, reauthorized PEPFAR for five more years, increasing the budget between 2008 and 2013 to more than US$48 billion [7].

An increasing number of published studies have supported the successes of PEPFAR. There has been a well-documented increase in individuals receiving HIV care in locations receiving PEPFAR funding [8], and several studies have suggested local decreases in mortality where HIV services have been scaled up [9,10]. Most recently, Bendavid and Bhattacharya [11] demonstrated that PEPFAR focus countries appear to be doing significantly better than non-focus countries when analyzing HIV-specific health outcomes, including HIV-related mortality and persons living with HIV. However, the question as to whether PEPFAR has had meaningful impact on the broader health care system remains unanswered.

In addition to the three primary goals of HIV prevention, treatment and support, the PEPFAR programme, particularly its reauthorization, also aims to increase health care capacity and reform in countries receiving PEPFAR funds [4,12,13]. This is of utmost importance as most middle- and low-income countries, such as the 15 PEPFAR focus countries, suffer from inadequate health sector supplies, infrastructure and human resources [14,15]. While many disease-specific (otherwise known as vertical) programmes have been successful in yielding improved outcomes related to the disease entities they are designed to address [16,17], they are often not nearly as effective in changing policy and reforming health systems [18,19].

Furthermore, many authors have argued that vertical programmes do not improve, and can actually harm, the overall health status of a population. It has been postulated that vertical programmes: (1) do not produce significant spillover in terms of additional resources for addressing other diseases and/or programmes within the health care sector [20]; (2) may displace funding resources from other important programmes [21]; and (3) can create an internal "brain drain" by diverting intellectual resources and human capacity from lower paying government jobs to higher paying vertical programme positions [22]. As a result, the World Health Organization (WHO) has instead advocated for an alternative approach that funds health care through a sector-wide approach[14].

A sector-wide approach represents a nationally based effort to increase health sector coordination, national leadership and ownership, and strengthen countrywide management and health care delivery systems [18]. In theory, such an approach reduces duplication of efforts, lowers transaction costs, increases equity and sustainability, and improves aid effectiveness and health sector efficiency [23]. However, for donor organizations and governments, a sector-wide approach is often less attractive because countries receiving funds are prioritizing programme funding based on a national health strategy, rather than on the donors' interests. This results in significantly less donor control when compared with traditional bilateral funding mechanisms.

In a 2007 report, the Institute of Medicine (IOM), the body charged with monitoring PEPFAR, expressed the possibility that a vertical programme, such as PEPFAR, can improve overall national health [24]. The report stated that explicit intervention priorities, such as HIV/AIDS, can be used to drive desired improvements into the health system [24]. This same position - that the scale up of HIV care and treatment, if designed and implemented appropriately, can have broad health benefits - was taken by El-Sadr and Abrams [25]. They make the logical argument that with such large sums of money being directed towards HIV, it would be necessary to improve infrastructure, expand the health care workforce and strengthen health systems, leading to improved health outcomes more broadly.

However, there is no evidence to date suggesting that PEPFAR has yielded any significant changes in overall mortality or other national health indicators that are not explicitly HIV related [26]. This is a critical gap in our understanding of the effects of this programme. The IOM report states that the "benefits and unintended consequences [of PEPFAR] will not be fully appreciated if the initiative is evaluated only with respect to HIV/AIDS targets .... Measures of this impact need to include workforce and infrastructure, as well as other health outcomes, such as infant mortality and all cause mortality" [24].

The purpose of this study is to assess the association between PEPFAR funding and changes in a broad range of health indicators among 46 countries in the WHO African Region.

## Methods

### Study type

This study was a retrospective analysis of publicly available health indicators from 46 African countries.

### Data source

PEPFAR focus countries were identified based on their designation by the Office of the United States Global AIDS Coordinator http://www.pepfar.gov. The WHO Statistical Information System (WHOSIS) was utilized as the sole data source. All data are publicly available through the WHO website http://www.who.int/whosis/data/Search.jsp.

### Data collection

Data collection was completed in September 2008. A search of health indicators by year was performed using the WHOSIS database for all countries within the WHO Africa Region. Socio-economic and demographic indicators were excluded from the initial database search. All indicators related to mortality, morbidity, human resources, access to care and health resources from the years 2000 and 2006 were selected as these were the two years in which data were available. Indicators were further limited by sex: when male, female and both sexes were included as separate indicators, only "both sexes" was included in the final analysis. Twelve additional indicators that dealt purely with health care financing were eliminated.

### Data analysis

Data was compiled onto an Excel spreadsheet (Microsoft Excel, Microsoft Corporation, Redmond, WA) and translated into a native SAS format using DBMS/Copy^® ^(Dataflux Corporation, Cary, NC). Analyses were conducted using SAS version 9.1 (SAS Institute, Cary, NC). Descriptive statistics were calculated for all indicators. When appropriate, numerical variables were compared using the non-parametric Wilcoxon rank sum test or the non-parametric signed-rank test, and are reported as medians with interquartile ranges (IQRs). No adjustment was made for multiple comparisons.

Countries were divided into PEPFAR focus and non-focus countries. Using the year 2000 as the baseline comparator, a fractional change was calculated for each indicator in each country across the WHO Africa Region. This allowed each country to serve as its own baseline control. A negative fractional change indicates an improvement in a given indicator (e.g., a decrease in mortality). Likewise, a positive fractional change suggests a worsening health indicator (e.g., an increase in tuberculosis prevalence).

To be consistent with this definition, the fractional change for the following health indicators were reversed (a negative value was made positive and vice versa): life expectancy at birth; neonates protected at birth against neonatal tetanus; one year olds immunized with meningococcal conjugate vaccine (MCV); one year olds immunized with three doses of diphtheria, tetanus and pertussis (DTP) vaccine; population with sustainable access to improved drinking water sources; population with sustainable access to improved sanitation; and TB detection rate under directly observed treatment, short course (DOTS). For example, the life expectancy in Rwanda between 2000 and 2006 increased from 46 to 52 years. The fractional change is calculated at 0.13, but changed to -0.13 to reflect an improvement.

A second analysis comparing PEPFAR focus and non-focus countries was performed utilizing a slightly different set of 29 non-focus countries in order to maintain some consistency with the recently published work of Bendavid and Bhattacharya [11].

### Study approval

The study was approved as exempt by the Human Subjects Committee (IRB) of the Los Angeles Biomedical Research Institute at Harbor-UCLA Medical Center.

## Results

The WHO Africa Region is comprised of 46 countries, 12 of which were given PEPFAR focus country designation by the Office of the United States Global AIDS Coordinator. The remaining 34 are non-focus countries. One hundred and forty-nine health indicators were found in the initial database search, most of which were missing data points. Of the indicators with complete or nearly complete data sets, 14 met inclusion criteria as defined in the Methods section.

### WHO Africa Region

**Figure 1:**
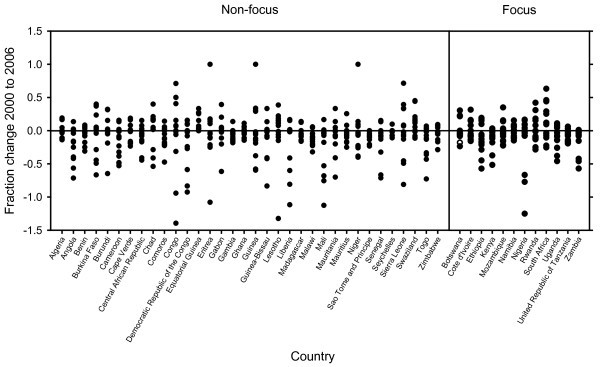
**Graphical Display of fractional changes in all reported health indicators for each PEPFAR focus and non-focus country**. Improvements in health indicators are indicated by negative fractional changes (see text).

Figure 1 (composite graph) represents the fractional change in all utilized health indicators across all countries in the WHO Africa Region. Although a visual inspection reveals no clear trend towards improving or worsening health indicators within the region as a whole, a statistical analysis shows a modest, but statistically significant 3.5% average worsening over all health indicators.

**Figure 2:**
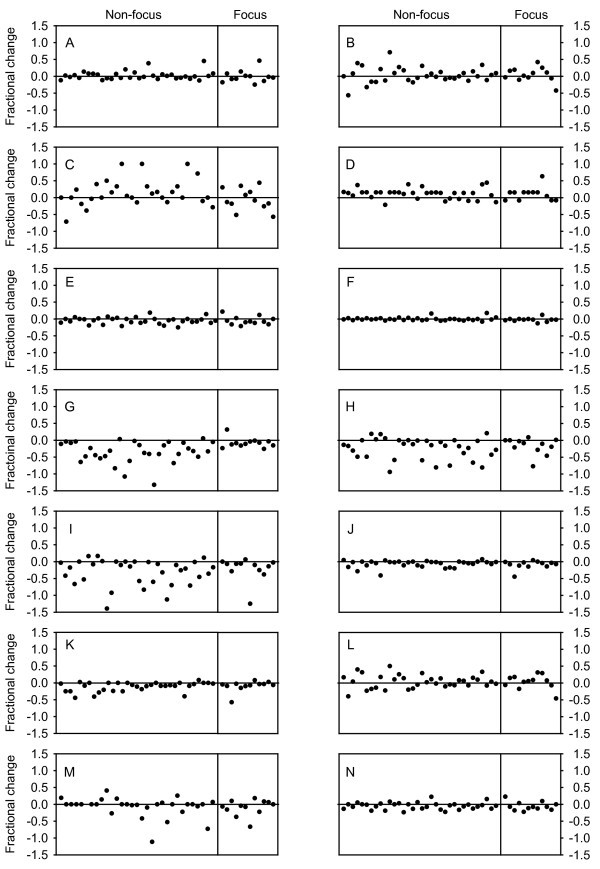
**Graphical display of fractional changes in each of the 14 health indicators considered, with each plotted point representing a single country**. As in Figure 1, countries to the left of the vertical line in each panel are non-focus countries and those to the right of the line are PEPFAR focus countries. The health indicators are: (a) adult mortality rate; (b) deaths due to TB among HIV-negative people; (c) deaths due to TB among HIV-positive people; (d) incidence of TB; (e) infant mortality rate; (f) life expectancy at birth; (g) neonates PAB against neonatal tetanus; (h) one year olds immunized with MCV; (i) one-year-olds immunized with three doses of DTP; (j) population with sustained access to improved drinking water; (k) population with sustained access to improved sanitation; (l) prevalence of TB; (m) TB detection rate under DOTS; and (n) under-five mortality rate

However, when each indicator is analyzed independently (Figure 2), it appears that most are actually improving. In fact, nine of the 14 health indicators have a negative median value and eight of these are statistically significant (Table 1). The range of improvement varies from a 1.6% fractional improvement in life expectancy at birth to a 19.7% gain in neonates protected at birth (PAB) against neonatal tetanus. The remaining five indicators all have a median fractional change that may indicate some worsening in the health indicator, but none are statistically significant.

**Table 1:** Overall changes in health indicators in the WHO Africa Region

Health indicator	Median fractional change*		p value*^+^*
Adult mortality rate	-0.007	(-0.064, 0.069)	0.919
Deaths due to TB, HIV negative	0.008	(-0.087, 0.162)	0.204
Deaths due to TB, HIV positive	0.000	(-0.135, 0.333)	0.112
Incidence of TB	0.137	(-0.028, 0.155)	< 0.001
Infant mortality rate	-0.052	(-0.115, 0.000)	0.002
Life expectancy at birth	-0.016	(-0.035, 0.000)	0.035
Neonates PAB against neonatal tetanus	-0.197	(-0.428, -0.063)	< 0.0001
One year olds immunized with MCV	-0.143	(-0.431, 0.000)	< 0.0001
One year olds immunized with 3 doses of DTP	-0.142	(-0.455, -0.012)	< 0.0001
Population with sustainable access to improved drinking water sources	-0.031	(-0.111, 0.000)	< 0.0001
Populations with sustainable access to improved sanitation	-0.071	(-0.148, 0.000)	< 0.0001
Prevalence of TB	0.046	(-0.074, 0.153)	0.180
TB detection rate under DOTS	0.000	(-0.156, 0.045)	0.111
Under-5 mortality rate	-0.064	(-0.130, 0.000)	0.001
All indicators	0.035	(-0.042, 0.156)	< 0.0001

* Each fractional change is normalized to the reported value in 2000. Values are shown as medians with interquartile ranges (IQRs). The number of countries in the region reporting each health indicator varies from 43 to 46.

^+ ^The p value addresses the question of whether the observed fractional change is statistically significantly different than zero, as assessed by the Wilcoxon signed rank test.

### Comparison by PEPFAR focus country designation

A comparison of PEPFAR focus countries with the non-focus countries is found in Table 2. Eleven of the 14 health indicators have negative median values among the PEPFAR focus country group, and eight of the 14 health indicators have negative median values among the non-focus group. Yet, when we compare the PEPFAR focus countries and the non-focus countries, a non-significant p value is noted among all of the health indicators, with the exception of neonates PAB against neonatal tetanus.

**Table 2:** Comparison of changes in health indicators in PEPFAR focus and non-focus countries

Health indicator	Fractional change from 2000 to 2006*				p value**
	PEPFAR focus countries†		Non-focus countries‡		
Adult mortality rate	-0.029	(-0.111, 0.046)	0.002	(-0.058, 0.069)	0.348
Deaths due to TB, HIV negative	0.057	(-0.048, 0.178)	0.000	(-0.111, 0.146)	0.576
Deaths due to TB, HIV positive	-0.109	(-0.220, 0.236)	0.050	(-0.037, 0.333)	0.139
Incidence of TB	0.153	(-0.082, 0.156)	0.137	(0.012, 0.155)	0.920
Infant mortality rate	-0.079	(-0.139, 0.013)	-0.041	(-0.108, 0.000)	0.616
Life expectancy at birth	-0.021	(-0.048, 0.000)	-0.007	(-0.035, 0.019)	0.187
Neonates PAB against neonatal tetanus	-0.099	(-0.157, -0.037)	-0.328	(-0.486, -0.078)	0.011
One year olds immunized with MCV	-0.094	(-0.248, 0.000)	-0.157	(-0.490, 0.000)	0.507
One year olds immunized with 3 doses of DTP	-0.085	(-0.269, -0.042)	-0.173	(-0.578, -0.010)	0.460
Population with sustainable access to improved drinking water sources	-0.060	(-0.130, -0.017)	-0.022	(-0.111, 0.000)	0.367
Populations with sustainable access to improved sanitation	-0.053	(-0.092, -0.028)	-0.085	(-0.207, 0.000)	0.597
Prevalence of TB	0.064	(-0.066, 0.162)	0.038	(-0.079, 0.153)	0.851
TB detection rate under DOTS	-0.056	(-0.190, 0.072)	0.000	(-0.097, 0.000)	0.659
Under-5 mortality rate	-0.077	(-0.144, 0.017)	-0.038	(-0.130, 0.000)	0.698
All indicators	0.038	(-0.073, 0.148)	0.033	(-0.033, 0.167)	0.301

* Each fractional change is normalized to the reported value in 2000. Values are shown as medians with interquartile ranges (IQRs).

**† **PEPFAR focus countries are Botswana, Cote d'Ivoire, Ethiopia, Kenya, Mozambique, Namibia, Nigeria, Rwanda, South Africa, Uganda, United Republic of Tanzania, Zambia. All PEPFAR focus countries reported each of the above health indicators.

**‡ **The non-focus countries are Algeria, Angola, Benin, Burkina Faso, Burundi, Cameroon, Cape Verde, Central African Republic, Chad, Comoros, Congo, Democratic Republic of the Congo, Equatorial Guinea, Eritrea, Gabon, Gambia, Ghana, Guinea, Guinea-Bissau, Lesotho, Liberia, Madagascar, Malawi, Mali, Mauritania, Niger, Sao Tome and Principe, Senegal, Seychelles, Sierra Leone, Swaziland, Togo, Zimbabwe. The number of non-focus countries reporting each health indicator varies from 31 to 34.

** The p value addresses the question of whether the median fractional change for each health indicator is statistically significantly different in PEPFAR focus and non-focus countries, as assessed by the Wilcoxon rank sum test.

Although both focus and non-focus countries showed fractional improvement in neonates PAB against neonatal tetanus, the non-focus countries actually performed significantly better than the PEPFAR focus countries (p value 0.011). A second analysis utilized the 29 non-focus countries found in the Bendavid and Bhattacharya paper and resulted in a similar trend.

### Country-level results

A country-level analysis utilizing all 14 health indicators can be found in Table 3 (PEFPFAR focus countries) and Table 4 (non-focus countries). Among PEPFAR focus countries, all except for South Africa seemed to be trending towards improvement with a negative median fractional change. Four of these countries (Kenya, Uganda, Tanzania and Zambia) had statistically significant improvements ranging from 8.5% to 5.1%.

**Table 3:** Changes in health indicators among PEPFAR focus countries

Country	Median fractional change*		p value^+^
Botswana	-0.035	(-0.067, 0.000)	0.569
Cote d'Ivoire	-0.026	(-0.080, 0.147)	0.677
Ethiopia	-0.145	(-0.212, 0.100)	0.091
Kenya	-0.080	(-0.117, -0.024)	0.003
Mozambique	-0.034	(-0.148, 0.033)	0.358
Namibia	-0.018	(-0.100, 0.063)	0.455
Nigeria	-0.030	(-0.077, 0.088)	0.583
Rwanda	-0.048	(-0.126, 0.154)	0.946
South Africa	0.108	(-0.074, 0.291)	0.135
Uganda	-0.085	(-0.257, 0.044)	0.035
Republic of Tanzania	-0.065	(-0.159, -0.020)	0.005
Zambia	-0.051	(-0.154, 0.000)	0.002

* Each fractional change is normalized to the reported value in 2000. Values are shown as medians with interquartile ranges (IQRs). The number of health indicators reported by each country varies from 13 to 14.

^+ ^The p value addresses the question of whether the observed fractional change is statistically significantly different than zero, as assessed by the Wilcoxon signed rank test.

**Table 4:** Changes in health indicators among PEPFAR non-focus counties

Country	Median fractional change*		p value^+^
Algeria	-0.018	(-0.111, 0.045)	0.891
Angola	-0.099	(-0.396, 0.000)	0.019
Benin	-0.031	(-0.080, 0.000)	0.110
Burkina Faso	0.026	(-0.286, 0.235)	1.000
Burundi	0.000	(-0.021, 0.024)	0.945
Cameroon	-0.171	(-0.481, -0.011)	0.020
Cape Verde	0.000	(-0.178, 0.012)	0.244
Central African Republic	-0.045	(-0.141, 0.000)	0.077
Chad	0.049	(0.016, 0.175)	0.497
Comoros	-0.120	(-0.207, 0.014)	0.027
Congo	0.034	(-0.059, 0.407)	0.622
Democratic Republic of the Congo	-0.022	(-0.271, 0.095)	0.204
Equitorial Guinea	0.033	(0.000, 0.153)	0.004
Eritrea	-0.076	(-0.213, 0.141)	0.542
Gabon	0.000	(-0.024, 0.033)	1.000
Gambia	-0.051	(-0.136, 0.000)	0.024
Ghana	-0.030	(-0.111, 0.017)	0.186
Guinea	-0.087	(-0.188, 0.291)	0.502
Guinea-Bissau	-0.019	(-0.100, 0.017)	0.267
Lesotho	0.094	(-0.059, 0.160)	0.268
Liberia	-0.008	(-0.412, 0.000)	0.131
Madagascar	-0.054	(-0.091, 0.000)	0.257
Malawi	-0.108	(-0.200, -0.048)	0.002
Mali	-0.045	(-0.529, -0.028)	0.008
Mauritania	-0.006	(-0.091, 0.000)	0.275
Mauritius	-0.035	(-0.102, 0.000)	0.232
Niger	-0.056	(-0.246, 0.064)	0.268
Sao Tome and Principe	-0.074	(-0.133, -0.008)	0.004
Senegal	-0.053	(-0.128, 0.134)	0.588
Seychelles	-0.006	(-0.071, 0.000)	0.219
Sierra Leone	-0.022	(-0.127, 0.331)	0.903
Swaziland	0.086	(-0.017, 0.176)	0.043
Togo	-0.045	(-0.333, 0.009)	0.043
Zimbabwe	-0.035	(-0.135, 0.044)	0.194

* Each fractional change is normalized to the reported value in 2000. Values are shown as medians with interquartile ranges (IQRs). The number of health indicators reported by each country varies from 11 to 14.

^+ ^The p value addresses the question of whether the observed fractional change is statistically significantly different than zero, as assessed by the Wilcoxon signed rank test.

In the non-focus country group, 25 countries have negative median values and nine positive median values. Eight countries demonstrate statistically significant improvements (Angola, Cameroon, Comoros, Gambia, Malawi, Mali, Sao Tome and Principe, and Togo) with medians ranging from 17.1% to 4.5%; two (Equitorial Guinea and Swaziland) have statistically significant worsening with fractional changes ranging from 8.6% to 3.3%.

## Discussion

To our knowledge, this is the first study to compare PEPFAR focus and non-focus countries, using non-HIV-specific national health indicators, since the inception of the programme. An initial glance at the data suggests that PEPFAR focus and non-focus countries are performing similarly with regard to multiple health indicators. While overall, most countries in the WHO Africa Region appear to be improving, the pace of improvement is nearly the same in both PEPFAR focus and non-focus countries.

If PEPFAR was designed as a vertical programme with no intention to improve health on a broader scale, our findings could reflect the fact that HIV is not the leading cause of mortality, or that HIV does not represent a large burden of disease in many of these countries (e.g., approximately 2.1% and 3.1% of the population is infected with HIV in PEPFAR focus countries Ethiopia and Rwanda, respectively) [2]. As a result, even a significant effect on HIV mortality (and HIV-associated health indicators in general) might not be noticeable in a general population analysis.

Using that same logic, however, we would expect to see potentially large gains in broad categories, such as all-cause mortality (infant, child and adult), vaccination rates, and decreasing incidence of highly prevalent diseases (e.g., tuberculosis) in countries with high HIV prevalence rates. Interestingly enough, South Africa, where the HIV prevalence rate is 18.8%, is the only PEPFAR focus country with a median value that would seem to indicate an overall worsening of health care indicators (although not statistically significant).

As clearly stated by the OGAC and the IOM, PEPFAR strives to not only improve HIV prevention, treatment and care, but also to improve the health system as a whole, by boosting infrastructure, training and supplies, and increasing public confidence in the health care system of many developing countries [4,24]. The WHO data we have analyzed, which incorporates the first three years of the PEPFAR programme (2003 to 2006), indicates that PEPFAR may not yet be successful in achieving the latter goals.

On the other hand, we do not see any evidence of PEPFAR having a deleterious effect. If supplies, attention and task shifting were employed in a way that resulted in decreased rates of immunization, clinic staffing or health care resources, we might expect to see a trend towards worsening non-HIV-specific health indicators. In fact, our analysis shows that 11 of the 12 PEPFAR focus countries are actually moving in the right direction with respect to multiple health indicators.

We must also question whether PEPFAR might actually be an effective approach to HIV/AIDS in Africa and, if so, why we might obtain the results presented here. First, has there been enough time for PEPFAR to make a difference? The 2006 data from WHO was likely collected early in the year (if not in 2005), and PEPFAR, although it was started in 2003, was not in full operational force until 2004. There may not have been adequate time for allocated monies to have reached the local agencies.

Second, it is possible that the monetary sum represented by PEPFAR, although very large, is still not enough. In low-income countries, like those in the WHO Africa Region, billions of dollars may still not be enough when dealing with such large deficits in health care infrastructure, personnel and resources.

Third, perhaps money is not the driving factor for change. Additional factors, such as political corruption, poor utilization of resources, problems with aid disbursement, lack of education, and a "donor-driven" rather than "owner-driven" agenda, may be obstacles too significant to overcome even with significant sums of money.

This study should be seen as a step in the overall evaluation of PEPFAR. A more comprehensive re-evaluation, using similar, and preferably many more, health indicators after several more years of the programme, would be an appropriate next step. It is important that outcomes, such as hospitalization, morbidity and mortality, are utilized in future analyses, including IOM PEPFAR evaluations. While the goals set for antiretroviral therapy and caring for those infected with HIV provide important early markers, and aid in the motivation of staff, true outcome data will determine the success of this programme and answer the question of whether a vertical programme can have broader effects on public health.

### Limitations

While the data that we have presented represents the best publicly available information we are aware of, we note that many data points were unchanged between 2000 and 2006. The lack of any apparent change over this six-year period may be accurate, or it may reflect a lack of data quality, or even a country simply reporting old numbers to address newly requested data points in the absence of new information. It is likely very difficult to conduct an accurate national survey for many of these health indicators, especially in developing countries. It is unclear whether PEPFAR focus and non-focus countries face similar challenges in data collection, or what bias is likely to result from poor data collection procedures.

In addition, this study does not account for non-PEFAR health sector foreign assistance, and does not try to quantify what would have happened without PEPFAR assistance in focus countries. Non-PEPFAR health sector funding may have a significant impact in both PEPFAR focus and non-focus countries, but in this study we did not attempt to quantify the amount or effect of non-PEPFAR funding.

While it is true that specific PEPFAR-funded care surely saves some individual lives, our results failed to demonstrate an inter-country association between PEPFAR funding and a variety of health status indicators. One possible explanation is that health indicators might have fallen without PEPFAR funding. However, such a fall was not observed in the non-PEPFAR-funded countries, so we were unable to find empirical support for that explanation.

Another limitation in the available data is the small number of health indicators and the utilization of only two time periods. Ideally, we would like to use a richer set of health indicators, representing a wider variety of disease processes, health care services and encounters, and public health processes. The availability of only two measurements for each indicator is also a significant limitation. While we calculated a fractional change, there is no way to know how that change occurred during the six-year period from 2000 to 2006. The use of more high-frequency measurements would be highly beneficial for ongoing evaluations of the PEPFAR programme, and something that PEPFAR money should potentially support.

Finally, because of difficulty defining the relative importance of different health outcomes, all health indicators (and all countries) were weighted equally in the statistical analysis. This means, for example, that adult mortality rate was given equal importance to one year olds immunized with meningococcal conjugate vaccine. Similarly, a large country, South Africa with a population of 47.9 million, was given equal weight to Sao Tome and Principe, with its population of just over 200,000 [27]. However, to partially address this limitation, all analyses were further stratified by country and by health indicator.

## Conclusions

PEPFAR represents the largest single government effort to combat HIV/AIDS worldwide. Although its primary goals are HIV related, its secondary goals of improving health care resources, infrastructure and workforce as a means of improving overall health are much broader, and perhaps more important. However, our analysis of available WHO health indicators between 2000 and 2006 demonstrates no significant difference in improvement in PEPFAR focus countries when compared with non-focus countries. Further studies will be necessary to detect the association, if one exists, between PEPFAR funding and non-HIV-specific health outcomes.

## Competing interests

The authors declare that they have no competing interests.

## Authors' contributions

HD, TC, GS, and RL were involved in the development of the study concept. HD, TC and RL worked on study design. HD performed data collection. AK and RL assisted with data analysis. HD created the manuscript, with editing and revision by AK, TC and RL. All authors reviewed and agree with the findings in the final manuscript.
